# Development of casein‐based nanoencapsulation systems for delivery of epigallocatechin gallate and folic acid

**DOI:** 10.1002/fsn3.827

**Published:** 2019-01-24

**Authors:** Parisa Malekhosseini, Mehran Alami, Morteza Khomeiri, Sara Esteghlal, Abdo‐Reza Nekoei, Seyed Mohammad Hashem Hosseini

**Affiliations:** ^1^ Department of Food Science and Technology Gorgan University of Agricultural Sciences and Natural Resources Gorgan Iran; ^2^ Department of Food Science and Technology School of Agriculture Shiraz University Shiraz Iran; ^3^ Department of Chemistry Shiraz University of Technology Shiraz Iran

**Keywords:** EGGC, folic acid, nanoencapsulation, re‐combined casein micelle

## Abstract

In this work, binding characteristics of two hydrophilic nutraceutical models, namely epigallocatechin gallate (EGCG) and folic acid (FA), to sodium caseinate were studied by fluorimetry technique. EGCG‐loaded casein molecules were then converted to either re‐combined casein micelles (r‐CMs) or casein nanoparticles (CNPs). Binding stoichiometry of EGCG and FA was 0.81 and 1.02, respectively. As determined by DLS technique, the average particle size of r‐CMs prepared at 0.5% concentration was 66.2 nm. Thermal treatment (74°C, 20 s) had significant (*p* < 0.05) influence on the particle size of nanocarriers, but not nutraceutical loading. The average size of CNPs was larger than that of r‐CMs. The encapsulation efficiency (EE) of EGCG was 85%, and its ejection from the nanocarrier was less than 3% over 21 days. Alkaline conditions resulted in higher release of EGCG than acidic conditions. r‐CMs were more effective than CNPs during the protection of EGCG against heat‐induced degradation. TEM micrographs confirmed the formation of r‐CMs.

## 1 INTRODUCTION

Nutraceutical compounds, as the link between nutrition and medicine, have gained increasing attention in recent years. Epigallocatechin gallate (EGCG), the ester of epigallocatechin and gallic acid, is the main (60%–70%) catechin present in green tea (de Pace et al., 2013; Ru, Yu, & Huang, 2010). EGCG has hydrophilic properties and hence is water‐soluble. EGCG can be utilized to decrease or control neurodegenerative and cardiovascular diseases as a result of antioxidant and anticancer properties (Dube, Nicolazzo, & Larson, 2010). Only around 0.1%–1.1% of total ingested EGCG is bioavailable for absorption (Dube et al., 2010; Holt, De Kruif, Tuinier, & Timmins, 2003). Limited bioavailability is due to low chemical stability and low permeability of intestine toward it (Dube et al., 2010; Haratifar, Meckling, & Corredig, 2014a; de Pace et al., 2013). Folic acid (FA) as a water‐soluble vitamin is another example of hydrophilic nutraceuticals. As a synthetic form of folate, FA is composed of pterin, p‐aminobenzoyl, and L‐glutamic acid (Liang & Subirade, 2010). FA has different functional properties and is particularly well known for preventing neural tube defects. Most folate derivatives are highly susceptible to oxygen, temperature, pH, and light during food processing (Madziva, Kailasapathy, & Phillips, 2005). Therefore, finding appropriate strategies to increase the chemical stability of hydrophilic bioactives is an active research area in food science (Assadpour, Maghsoudlou, Jafari, Ghorbani, & Aalami, 2016). However, for hydrophobic nutraceuticals, an increase in chemical stability as well as aqueous solubility (physical stability) is of concern (Mirpoor, Hosseini, & Nekoei, 2017).

Encapsulation technology may result in increasing the physicochemical stability and bioavailability of orally administered nutraceuticals through not only bioactive protection but also controlling the release rate at the absorption site (Esfanjani & Jafari, 2016; Katouzian & Jafari, 2016). Food‐grade carriers are the preferred choice mainly due to non‐toxicity (Hu et al., 2012). Different carriers have been used for delivery of EGCG and FA delivery such as nanolipid particles (Smith et al., 2010), chitosan nanoparticles (Dube et al., 2010), peptide‐chitosan complex nanoparticles (Hu et al., 2012), β‐lactoglobulin nanoparticle (Liang & Subirade, 2010; Shpigelman, Cohen, & Livney, 2012), nanoliposomes (de Pace et al., 2013) and O/W emulsion (Ru et al., 2010) biopolymer complexes (Madziva et al., 2005), and spray‐dried nanoemulsions (Assadpour & Jafari, 2017).

Casein Micelle (CM) is a nature‐made carrier for calcium and phosphate. Casein amounts 80% of total milk protein and is composed of various fractions including α_s1_‐, α_s2_‐, β‐, and κ‐caseins (Haratifar & Guri, 2017). The particle size distribution of CMs lies between 50 to 500 nm. CMs are stable against a variety of unit operations applied in food processing. In addition to the safety, bioavailability, and stability, CMs are an important source of essential amino acids, phosphate, and calcium (Haratifar, Meckling, & Corredig, 2014b; Semo, Kesselman, Danino, & Livney, 2007). CMs and casein nanoparticles (CNPs) have been suggested as suitable carriers for different nutraceuticals such as curcumin (Ghayour et al., 2019; Pan, Zhong, & Baek, 2013), vitamin D_3_ (Haham et al., 2012), polyphenols (Haratifar et al., 2014a,b), α‐tocopherol (Chevalier‐Lucia, Blayo, Gràcia‐Julià, Picart‐Palmade, & Dumay, 2011), quercetin (Ghayour et al., 2019), ω‐3 polyunsaturated fatty acids (Zimet, Rosenberg, & Livney, 2011), and anticancer drugs (Shapira, Assaraf, & Livney, 2010). Protection of EGCG against oxidation after encapsulation within natural CMs, separated from milk, was reported by Haratifar et al. (2014a). It was also reported that EGCG is bioaccessible after association with natural milk casein. The bioaccessibility was independent from casein digestion level (Haratifar et al., 2014b). Casein micelles re‐combined from sodium caseinate can be used in a similar way of natural CMs. The most important feature of re‐combined casein micelles (r‐CMs) over natural CMs is the tailor‐made particle size. Moreover, the binding (entrapment) process of bioactives is performed prior to the re‐assembling process. Therefore, higher encapsulation efficiencies are expected. The clearance rate of nanocarriers (<100 nm) by the reticuloendothelial system in liver and spleen is slower than that of larger carriers (de Pace et al., 2013).

The application of r‐CMs in the encapsulation of hydrophobic nutraceuticals has been studied already by Semo et al. (2007) and Zimet et al. (2011) and recently by Ghayour et al. (2019). To our knowledge, there is not any report about the application of re‐combined casein micelles (r‐CMs) or casein nanoparticles (CNPs) in the encapsulation of hydrophilic nutraceuticals. Both EGCG and FA have the ability to make molecular complexes with globular proteins. The first objective of this work was to study the binding properties of EGCG and FA to natively unfolded sodium caseinate. After that, we tried to make an insight into the potential application of casein‐based carriers in the encapsulation and protection of the nutraceutical model with higher sensitivity (i.e., EGCG). Therefore, after characterization of the binding properties of individual molecules (not a mixture of them) to sodium caseinate using fluorimetry technique, the EGCG‐loaded molecular complexes were converted to r‐CMs and CNPs, because of higher susceptibility of EGCG than FA and its lower binding affinity to sodium caseinate (discussed later) and then subjected to heat treatment and different pH conditions to check for the efficacy of nanocarriers.

## 2 MATERIALS AND METHODS

### 2.1 Materials

Sodium caseinate, EGCG, and folic acid (FA) were purchased from Sigma‐Aldrich Co. (St. Louis, MO, USA). HCl, NaOH, CaCl_2_, K_2_HPO_4_, Na_2_HPO_4_, tripotassium citrate (K_3_C_6_H_5_O_7_), and sodium azide (NaN_3_) were obtained from Merck Co. (Darmstadt, Germany).

### 2.2 EGCG and folic acid binding to sodium caseinate

Binding of EGCG and FA to sodium caseinate was characterized at pH 7.4 through measuring the binding‐induced quenching of the intrinsic fluorescence of the tryptophanyl residues of sodium caseinate fractions. For some globular proteins like beta‐lactoglobulin, the binding of ligands to the main binding site of carrier is pH‐dependent (Hosseini, Emam‐Djomeh, Sabatino, & Van der Meeren, 2015; Mirpoor, Hosseini, & Yousefi, 2017). However, this behavior is not generally observed for natively unfolded proteins (like sodium caseinate) and when polyphenolic ligands are applied. The applied method for re‐combining the micellar structures (section 2.3) was actually a simulation of Golgi system of mammalian cells which could result in the formation of structures with similar properties to native casein micelles at a neutral pH range (Zimet et al., 2011). Therefore, binding studies were performed at pH values required for the subsequent re‐combining process. Briefly, sodium caseinate dispersion was prepared in phosphate buffer (10 mM, pH 7.4) and hydrated for 12–15 hr. The dispersion was then centrifuged at 3500 g and 25°C for 25 min. The supernatant was collected, and its absorbance was measured at 278 nm. The concentration (*c*) of sodium caseinate was determined using the Beer–Lambert equation.

(1) A=εbc

where *A* is the absorbance; *ε* is extinction coefficient (0.81 L/g.cm for casein), and *b* is the light pass length. The concentration of clump‐free sodium caseinate dispersion was adjusted to 5 μM using phosphate‐buffered solution. A Varian Cary Eclipse fluorescence spectrophotometer was used to measure the binding‐induced quenching. Excitation was performed at 278 nm, and emission spectrum was recorded at 300–550 nm. After recording the emission spectrum of protein dispersion in the absence of ligands, sequential injections (2 μl) of either EGCG or FA solutions (dissolved in phosphate buffer in 10 mM concentration) were performed during 2‐min intervals. After each injection, a new spectrum was recorded. Injections were continued until the amount of ligand solution in the quartz cell reached to 34 μl. In order to remove the dilution effect, a blank experiment was performed using ligand‐free buffer solution. Binding parameters were determined using Equation (2).

(2) $$\text{Log}\left[\left(F_{0}-F_{i}\right)/F_{i}\right]=\text{log}K_{b}+n\text{log}\left[\mathrm{ligand}\right]$$

where *F*_0_ is the fluorescence intensity of control sodium caseinate dispersion (i.e., in the absence of EGCG and FA); *F*_i_ is the fluorescence intensity of sodium caseinate dispersion after i_th_ injection of either EGCG or FA (i.e., ligands); *K*_b_ is the binding constant (M^−1^); and *n* is the binding stoichiometry (ligand to casein molar ratio at saturation). To determine both binding parameters, Log [(*F*_0_ *− F*_i_)/*F*_i_] was linearly plotted against log EGCG (or log FA) concentration (log [*ligand*]). The slope and intercept were considered as *n* and log *K*_b_, respectively.

### 2.3 Formation of casein‐based nanocarriers

Re‐combined casein micelles (r‐CMs) at 0.5% (w/v) were prepared using the method described by Zimet et al. (2011) with slight modifications. Briefly, sodium caseinate dispersion (1% w/v) was prepared and hydrated at 4°C for 12–15 hr. To start the re‐combining process, 2 ml of tripotassium citrate (0.2 M), 12 ml of dipotassium phosphate (0.04 M), and 10 ml of calcium chloride (0.04 M) were added to 100 ml of protein dispersion at 4°C under stirring. Then, 1.25 ml of dipotassium phosphate (0.04 M) and 2.5 ml of calcium chloride (0.04 M) were added 8 times at 15‐min intervals. To complete the assembling process, distilled water was added to the protein dispersion to reach the final volume of 200 ml. During the process, pH was adjusted to 6.7–7.0.

Casein nanoparticles (CNPs) were actually nanoscale aggregates of casein fractions developed due to relatively low solubility in water. The main differences between the method of formation of CNPs and r‐CMs were the preparation temperature and not utilization of hydrated salts. In other words, CNPs were prepared at 25°C by dispersing sodium caseinate powder in deionized water. Hydrated salts were not utilized for the preparation of CNPs. In all samples, sodium azide (0.03% w/v) was used as an antimicrobial agent.

### 2.4 Particle size and distribution

A dynamic light scattering instrument (Nanotrac Wave, Microtrac, Montgomeryville, PA, USA) was used at 25°C to determine the volume‐weighted average particle size and distribution. Distilled water was used as the blank. Particle size distribution (Span) was calculated according to the Equation (3):

(3) $$\text{Span}=\left(D_{v0.9}-D_{v0.1}\right)/D_{v0.5}$$

where *D*_v0.9_, *D*_v0.1_, and *D*_v0.5_ are the diameters where 90%, 10%, and 50% of population lie below them, respectively.

### 2.5 Effect of heat processing

Thin layers of r‐CM and CNP were subjected to heat treatment (74°C for 20 s). The effect of thermal processing was determined by measuring the particle size as described already (section 2.4).

### 2.6 EGCG encapsulation

Epigallocatechin gallate was added to the protein dispersion at 1:1 molar ratio prior to the formation of r‐CM or CNP at 0.5% (w/v) concentration. EGCG‐loaded carriers were prepared in a similar manner as described in Section 2.3. A protein‐free control sample was also prepared.

### 2.7 Encapsulation characteristics

#### 2.7.1 Particle size distribution

A DLS instrument was used to determine the particle size distributions of EGCG‐loaded carriers before and after performing heat treatment.

#### 2.7.2 EGCG stability against heat‐induced degradation

An enhanced shelf‐life stress test performed at 45°C was used to estimate the protection conferred to the nanoencapsulated EGCG. Briefly, a shaker incubator was utilized for heating the EGCG‐loaded delivery systems (0.5% w/v r‐CM or CNP) at 45°C for 1 month. The amounts of the remained EGCG were then measured as a function of time. Sample containers were covered by aluminum foil to prevent from light‐induced degradation. To determine the amount of the remained EGCG, loaded ligand could not be easily extracted from the carrier (due to the water solubility of EGCG). Moreover, casein and EGCG both absorb light at 274 nm (Section 2.7.3). Therefore, it was postulated that the measured absorbance was a consequence of both EGCG and casein UV absorptions. Casein is a heat‐stable protein. Therefore, any change (reduction) in the absorbance could be attributed to the EGCG degradation. However, a blank (EGCG‐free CNP or r‐CM dispersions) was prepared and treated in a similar manner in order to determine any possible changes in UV‐light absorption of casein as a result of long‐term heat treatment. Another blank was a solution of EGCG in distilled water. To estimate the stability conferred to EGCG, the differences between the absorbance values of EGCG‐loaded and EGCG‐free delivery systems were plotted as a function of time.

#### 2.7.3 Encapsulation efficiency

Free (unloaded) EGCG molecules were separated from loaded ones using ultrafiltration method. Briefly, 4 ml of EGCG‐incorporated carriers was loaded onto Amicon ultrafiltration unit (10 kDa, Merck Millipore Ltd., Dublin, Ireland) and centrifuged at 3000 *g* and 25°C for 30 min. The amount of free EGCG was determined through measuring the absorbance of permeate at 274 nm and then calculating the concentration using a standard curve (Abs = 8.995x[*EGCG*] + 0.0173; *R* ^*2*^ = 0.99) prepared by EGCG standard solutions ranging from 0.04 to 0.1 μM. Encapsulation efficiency (EE) was determined according to the Equation (4):

(4) $$\text{EE}\%=(m_{t}-m_{p}/m_{t})\times 100$$

where *m*_*t*_ and *m*_*p*_ are the amounts (mg) of EGCG present in the initial sample and in the permeate, respectively.

#### 2.7.4 Stability

Stability indicates the ability of a delivery system to retain the encapsulated compound inside. To determine the stability, the amounts of EE (section 2.7.3) were plotted as a function of time.

#### 2.7.5 Effect of pH on the encapsulation efficiency

The pH value of r‐CMs loaded with EGCG was adjusted to 5, 5.7, 6, 6.7, 7.5, 8.5, and 9.5 using either 0.1 N HCl or 1 N NaOH. The EE was then measured (Section 2.7.2) as a function of pH.

### 2.8 Transmittance Electron microscopy (TEM)

The structure and morphology of r‐CM prepared at 0.5% (w/v) were characterized by a transmission electron microscope (TEM, CM‐10, Philips, the Netherlands) using an acceleration voltage of 60 kV. A drop of sample was placed directly onto a carbon‐coated copper grid (mesh size 300) then allowed to be quickly dried. After that, the sample was negatively stained (using uranyl acetate 3%) and examined by TEM in a very cold box.

### 2.9 Statistical analysis

All experiments were performed in at least triplicate. A completely randomized design was used in this study. In binding analysis, ligand type was independent variable and binding parameters were dependent variables. In particle size analysis, comparison was performed among EGCG‐free carrier, EGCG‐loaded carrier, and those subjected to heat treatment. In stability tests, pH and storage time were independent variables, while EE was dependent one. In bioprotection test, storage time was independent variable and the absorbance (indicating EGCG degradation) was dependent variable. SPSS program (ver. 20) was used for statistical analysis. Differences were considered to be significant at *p* < 0.05.

## 3 RESULTS AND DISCUSSION

### 3.1 Binding characteristics

**Figure 1:**
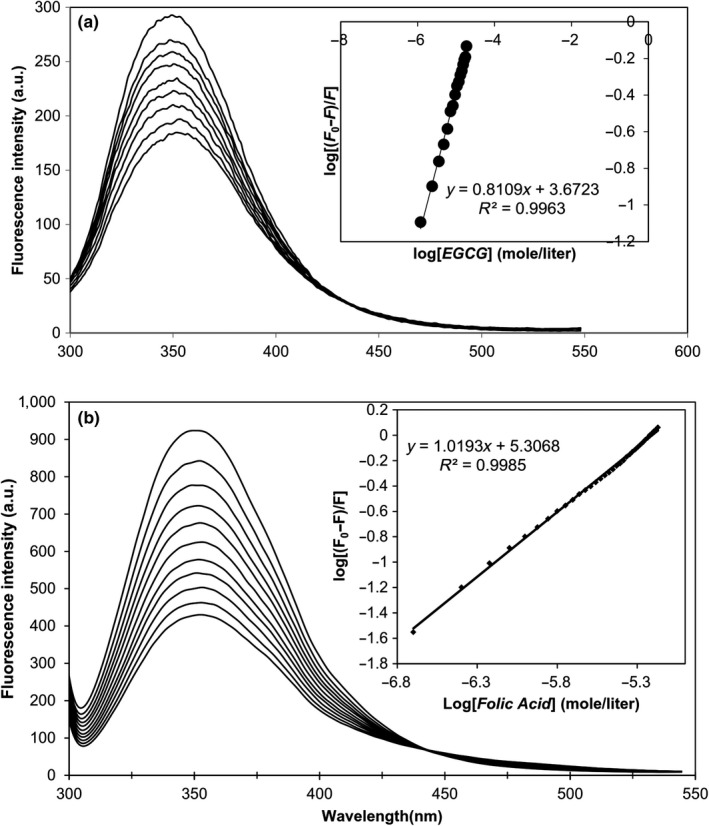
The fluorescence emission spectra of sodium caseinate (5 μM, pH 7.4) excited at 287 nm after successive injections of (a) EGCG and (b) FA (The inset shows the analysis of binding parameters)

Figure 1a,b depicts the emission spectra of casein as a result of sequential addition of EGCG and FA, respectively. The fluorescence properties of casein are attributed to the presence of aromatic amino acid (particularly tryptophan) residues in α‐casein (2), β‐casein (1), and κ‐casein (1) (Farrell et al., 2004). The models describing CM structure suggest that α‐ and β‐caseins are mainly located in the inner part of CM, while κ‐casein is dominantly located on the surface. Binding of ligand to protein generally results in decreasing (quenching) the intrinsic fluorescence. Decreasing the fluorescence intensity by increasing the amounts of EGCG and FA was a proof for the binding of these ligands to sodium caseinate. The linear plots of log[(*F*_0_‐*F*)/*F*] as a function of log[*ligand*] are shown as an insets in Figure 1. The amounts of *n* and *k*_b_ were 0.81 and 4.68x10^3^ M^−1^ for EGCG and 1.02 and 2.04 × 10^5^ M^−1^ for folic acid, respectively. Zimet et al. (2011) reported binding stoichiometry of 3.73 and association constant of 8.38 × 10^6^ M^−1^ for the binding of DHA to sodium caseinate. A binding constant of 5.24 × 10^4^ M^−1^ and a number of binding sites of 0.89 were reported by Benzaria, Maresca, Taieb, and Dumay (2013) for curcumin binding to native‐like phosphocaseins. It can be concluded that casein is capable of binding both lipophilic and hydrophilic nutraceuticals. The binding of lipophilic ligands is generally stronger than that of hydrophilic ones likely due to the prominent role of hydrophobic interactions. Binding of ligands to sodium caseinate is the main mechanism responsible for their encapsulation during the re‐combining process of micelles. Based on the fluorimetry results, EGCG:casein molar ratio of 1:1 was selected for the encapsulation process.

There are a limited number of binding locations in each protein molecule for the added ligands. In a mixture of ligands, different conditions such competitive binding or synergism may occur and therefore result in problems in data interpretation. Simultaneous addition of both ligands cannot be studied just by a single technique and a combination of different analytical techniques such as fluorimetry, NMR, and is required. To prevent from the aforementioned problems, the binding of EGCG and FA was separately studied with sodium caseinate. After obtaining the binding parameters, the nutraceutical compound with higher chemical sensitivity and also lower binding affinity of its complex with sodium caseinate (i.e., EGCG) was converted to either EGCG‐loaded r‐CMs or CNPs. We assumed that the encapsulation of FA leads to at least similar results to those of EGCG encapsulation, mainly due to the relatively higher chemical stability of FA and also its higher affinity (≈ 44 times) to make complexes with sodium caseinate.

### 3.2 EGCG protection

**Figure 2:**
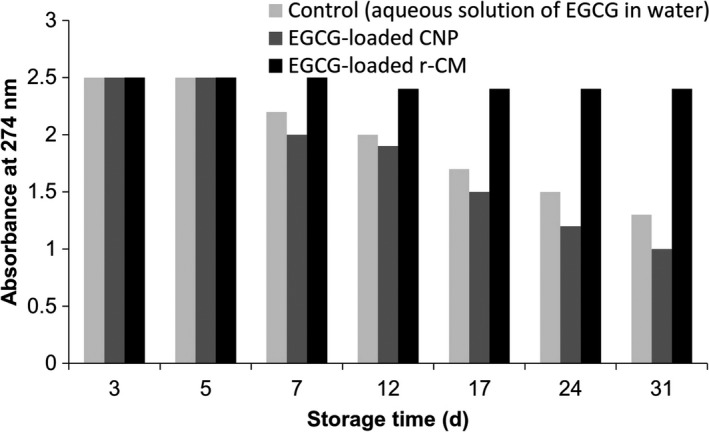
Effects of different casein‐based nanocarriers on the EGCG degradation as a function of time**;** CNP and r‐CM indicate casein nanoparticle and re‐combined casein micelle, respectively. A decrease in the absorbance indicates EGCG degradation (Section 2.7.5)

Figure 2 shows the rate of EGCG degradation as a function of time. Relatively similar patterns were observed for the blank solution of EGCG and the EGCG encapsulated by CNP. In both samples, degradation was initiated after 5 days. This result clearly indicated that EGCG binding to casein (i.e., EGCG delivery by CNP) is not sufficient for its effective protection, which can be attributed to the open structure of casein molecules. Shpigelman, Israeli, and Livney (2010) reported that thermally induced β‐lactoglobulin‐EGCG nanovehicles conferred significant protection to EGCG against oxidative degradation. Lower initial degradation rate (33 times) and slower degradation over 8 days (3.2 times) were observed for the encapsulated EGCG as compared to free EGCG (Shpigelman et al., 2012). Binding of EGCG to casein and then re‐combining to casein micelles led to a significant increase in the EGCG protection. Therefore, r‐CMs were more effective than CNP in the protection of EGCG. Zimet et al. (2011) reported that both CNP and r‐CM revealed significant protective effects on DHA against oxidation at 4°C. The differences between the results obtained in this study and those reported by Zimet et al. (2011) can be explained by the location of DHA and EGCG in CNP‐type carriers. Hydrophobic and hydrophilic ligands are located in the interior and the exterior of the protein structure, respectively. Binding to the exterior part might not have significant protection effect. Casein has also been successfully utilized for the protection of other nutraceuticals such as curcumin (Rahimi Yazdi & Corredig, 2012) and vitamin D_3_ (Haham et al., 2012).

### 3.3 Particle size analysis

Particle size distribution plays a key role in the physical properties of colloidal systems such as stability, turbidity, and rheology. Particle size is also effective on the bioavailability and organoleptic properties of colloidal food systems. The effects of various factors such as type of delivery system (r‐CM or CNP), EGCG loading, and heat treatment on the average particle size and distribution are shown in Table 1. The average particle size of r‐CMs prepared at 0.5% concentration was 66.2 nm. Shapira et al. (2010) investigated the encapsulation of a chemotherapeutic drug in β‐casein nanocarrier. They reported that the mean volume‐weighted diameter of nanocapsules prepared at 0.5% (w/v) was below 100 nm. Because of high physical stability and small particle size, r‐CMs prepared at 0.5% (w/v) were utilized for the encapsulation of EGCG. The average particle size of EGCG‐loaded r‐CMs before and after heat treatment is also reported in Table 1. The volume‐weighted mean diameter of non‐treated sample was 67.7 nm, indicating that EGCG loading had no significant (*p* > 0.05) effect on the particle size of delivery system. The increase in the Span might be attributed to the formation of protein–polyphenol complexes. Heat treatment increased the average particle size to 129.9 nm. Semo et al. (2007) reported an average particle size of 147 nm for r‐CMs loaded with vitamin D. Loading mitoxantrone (a chemotherapeutic drug) onto the β‐casein nanovehicles increased the average particle size from <100 to 150 nm (Shapira et al., 2010). A significant increase in the average particle size was observed after pasteurization. Similar results have been reported by Zimet et al. (2011) but in lower intensity. The particle size of DHA‐loaded r‐CM increased from 51.9 to 59.0 after heating at 74°C for 20 s. The authors concluded that heat treatment did not affect the particle size because of considerable thermal stability of casein (Zimet et al., 2011). Le, Saveyn, Hoa, and Van der Meeren (2008) measured the particle size of casein micelles in the absence and presence of whey protein using DLS and nanoparticle tracking analysis (NTA). The results of both techniques revealed that heating of casein micellar dispersions in the presence of whey proteins resulted in a bimodal distribution. The authors concluded that the increase in the average particle size is primarily a consequence of casein micellar aggregation followed by heat‐induced deposition of whey protein onto the casein micelles (Le et al., 2008). Therefore, in spite of high thermal stability of casein micelles, aggregation of micellar structures during heating is not beyond mind; hence, particle size increased for this reason. Heating results in the exposure of more hydrophobic patches and hence increasing the entropy of system (Esmaili et al., 2011). Hydrophobic interactions as the only entropy‐driven forces are likely responsible for the self‐association of r‐CMs. Moreover, the presence of EGCG may have an enhancing effect on increasing the particle size during heat treatment via protein–polyphenol interactions. The volume‐weighted mean diameter of CNP prepared at 1% (w/v) was 161.9 nm. Similarly, a value of 175 nm was reported by Semo et al. (2007). The smaller particle size of r‐CM than CNP could be a sign for the re‐combining process. The mixture of proteins in sodium caseinate is natively unfolded which might lead to larger effective volumes (higher scattering) of aggregated CNPs. Moreover, the compaction phenomenon as a result of different (particularly electrostatic and hydrophobic) interactions between various fractions of proteins and the hydrated salts during the re‐combining process might lead to a decrease in particle size (effective volume) of r‐CMs as compared to that of CNPs. Zimet et al. (2011) reported that the larger mean diameter of CNP (288.9 nm) than r‐CM (50–60 nm) was due to the longer processing time for the preparation of r‐CM at 4°C. These authors also concluded that the lower temperature (i.e., 4°C) applied in re‐combining micellar structures might result in weaker hydrophobic interactions and hence formation of smaller carriers (Zimet et al., 2011). An increase in the average particle size (245.7 nm) and Span (2.38) was observed after pasteurization of CNPs. The increase in the particle size can be attributed to the formation of small aggregates of casein fractions as a result of heat treatment. More uniformity of r‐CMs might lead to lower differences in the free surface energy and hence higher stability against heat treatment.

**Table 1:** Effects of carrier type, EGCG loading, and heat treatment on the average particle size and Span

Sample	Conc. (%w/v)	Heat treatment	*D*_v90_	*D*_v10_	*D*_v50_	Span	Mean diameter (nm)
r‐CM	0.50	−	132.5	27.95	39.5	2.64	66.2
r‐CM+EGCG	0.50	−	158.2	20.48	30	4.59	67.7
r‐CM+EGCG	0.50	+	223.5	33	56	3.40	129.9
CNP	1.00	−	417.0	93.5	161	2.01	161.9
CNP	1.00	+	717.5	140.5	242.5	2.38	245.7

r‐CM and CNP stand for re‐combined casein micelle and casein nanoparticle, respectively. *D*_v0.9_, *D*_v0.1_, and *D*_v0.5_ are the diameters (nm) where 90%, 10%, and 50% of population lie below them, respectively.

### 3.4 Nanoencapsulation characteristics

**Figure 3:**
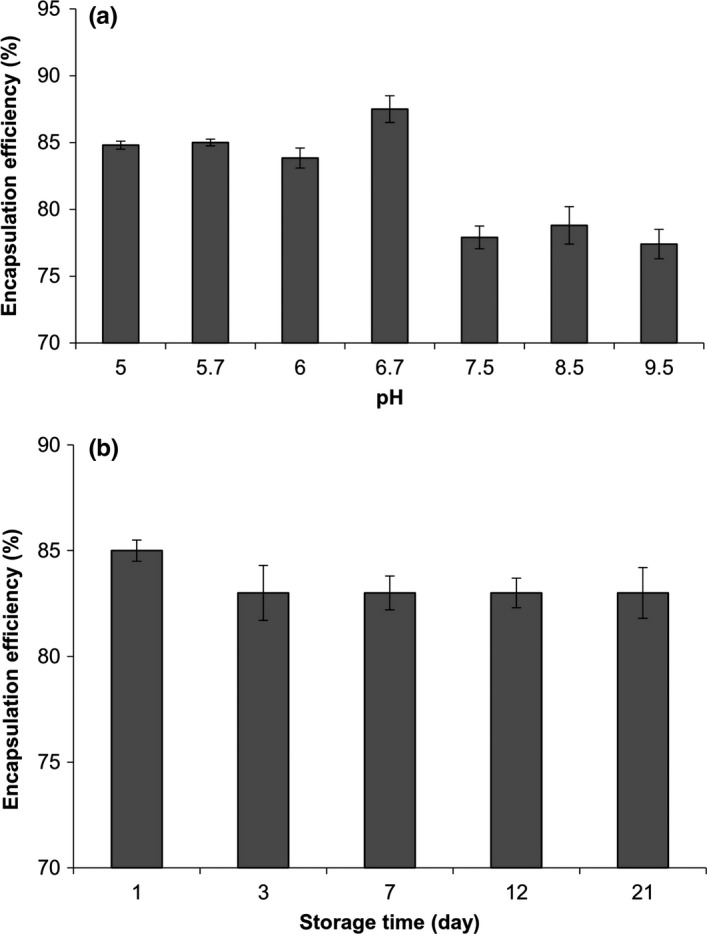
Changes in the encapsulation efficiency (EE%) of EGCG after loading into re‐combined casein micelles as a function of (a) pH and (b) storage time

The encapsulation efficiency (EE) of EGCG in r‐CM was 85%. Large amount of EE could be the result of EGCG binding to casein fractions through either hydrophobic interactions (between aromatic rings of phenol and hydrophobic amino acids in casein) or hydrogen bondings (between hydroxyl groups of EGCG and carbonyl groups of casein) (Hofmann et al., 2006). Semo et al. (2007) reported that the recovery efficiency of vitamin D_2_ after loading onto the casein micelles was 85%. A loading efficiency of 60%–70% has been reported for the EGCG encapsulation within the thermally induced β‐lactoglobulin nanocomplexes (Shpigelman et al., 2012). Changes in the EE as a function of pH are plotted in Figure 3a. Generally, an increase in the pH led to a decrease in the EE. This trend was attributed to the loose structure of casein micelles at higher pH values. The micellar structure of casein is due to various driving forces such as hydrogen bondings, electrostatic, and hydrophobic interactions. At higher pH values, the increase in the negative charge of casein results in more repulsion. The dissolution of calcium and phosphate ions in the serum as well as the increase in the electrostatic repulsion between the casein sub‐micelles result in a loosely bulk structure at alkaline pH values (Madadlou, Mousavi, Emam‐Djomeh, Sheehan, & Ehsani, 2009) and hence EGCG ejection from the nanocarriers.

Figure 3b depicts the amounts of EE as a function of time. During the first 3 days, a decrease in the EE was observed then remained constant until the end of the storage period (21 days). The observed decrease could be attributed to the ejection of loosely bound EGCG molecules from the carrier. This result showed that r‐CMs have high loading efficiency (i.e., they can be effectively used to deliver EGCG molecules over time). For each delivery system, changes in the matrix to reach lower free energies are the main reason responsible for the ejection of the entrapped compound over time (i.e., low loading efficiencies). The primary structure of casein has high amount of proline residues, which are responsible for the flexibility and adaptability of casein structure to different conditions (Holt et al., 2003).

### 3.5 Morphological studies

**Figure 4:**
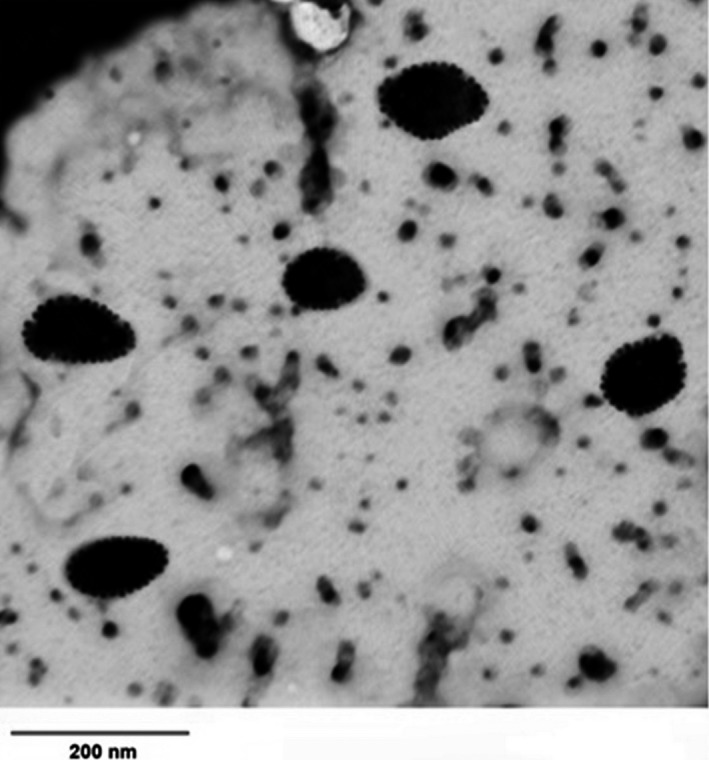
Transmittance electron microscopy image of nutraceutical‐free re‐combined casein micelles prepared at 0.5% (w/v)

Figure 4 shows the TEM image of r‐CM. Spherical nanoscale carriers having serrated edges were observed indicating likely the protruding of κ‐casein fraction.

## 4 CONCLUSIONS

Fluorimetry results revealed that sodium caseinate is able to bind hydrophilic EGCG and FA. EGCG‐loaded r‐CMs were developed using a hierarchical approach through first EGCG binding to sodium caseinate and then re‐combining of casein micelles. Heat treatment significantly increased the particle size. Large amount of EE was obtained; however, it was dependent on pH. The amount of EGCG ejection from the nanocarrier was not significant over time. The obtained delivery systems were of spherical shape. These results showed that r‐CMs can be used as a suitable carrier for hydrophilic nutraceuticals. More studies are required to determine whether casein‐based nanocarriers are able to increase the bioavailability of hydrophilic bioactive compounds. Simultaneous encapsulation of nutraceutical compounds is very interesting, provided taking into account their physicochemical compatibility and also the absorption kinetics into the body.

## ETHICAL REVIEW

The authors declare that they do not have any conflict of interest. Human testing and animal testing were unnecessary in our study.
